# Attenuation of Zika Virus by Passage in Human HeLa Cells

**DOI:** 10.3390/vaccines7030093

**Published:** 2019-08-20

**Authors:** Li Li, Natalie D. Collins, Steven G. Widen, Emily H. Davis, Jaclyn A. Kaiser, Mellodee M. White, M. Banks Greenberg, Alan D. T. Barrett, Nigel Bourne, Vanessa V. Sarathy

**Affiliations:** 1 Department of Pathology, University of Texas Medical Branch, Galveston, TX 77555, USA; 2 Department of Microbiology and Immunology, University of Texas Medical Branch, Galveston, TX 77555, USA; 3 Viral Disease Branch, Walter Reed Army Institute for Research, Silver Spring, MD 20910, USA; 4 Department of Biochemistry and Molecular Biology, University of Texas Medical Branch, Galveston, TX 77555, USA; 5 Department of Pediatrics, University of Texas Medical Branch, Galveston, TX 77555, USA; 6 Sealy Institute for Vaccine Sciences, University of Texas Medical Branch, Galveston, TX 77555, USA; 7 Institute for Human Infections and Immunity, University of Texas Medical Branch, Galveston, TX 77555, USA

**Keywords:** Zika virus, attenuation, flavivirus, HeLa cells, Zika virus prM, Zika virus NS1

## Abstract

Zika virus (ZIKV) is a mosquito-borne Flavivirus. Previous studies have shown that mosquito-transmitted flaviviruses, including yellow fever, Japanese encephalitis, and West Nile viruses, could be attenuated by serial passaging in human HeLa cells. Therefore, it was hypothesized that wild-type ZIKV would also be attenuated after HeLa cell passaging. A human isolate from the recent ZIKV epidemic was subjected to serial HeLa cell passaging, resulting in attenuated in vitro replication in both Vero and A549 cells. Additionally, infection of AG129 mice with 10 plaque forming units (pfu) of wild-type ZIKV led to viremia and mortality at 12 days, whereas infection with 10^3^ pfu of HeLa-passage 6 (P6) ZIKV led to lower viremia, significant delay in mortality (median survival: 23 days), and increased cytokine and chemokine responses. Genomic sequencing of HeLa-passaged virus identified two amino acid substitutions as early as HeLa-P3: pre-membrane E87K and nonstructural protein 1 R103K. Furthermore, both substitutions were present in virus harvested from HeLa-P6-infected animal tissue. Together, these data show that, similarly to other mosquito-borne flaviviruses, ZIKV is attenuated following passaging in HeLa cells. This strategy can be used to improve understanding of substitutions that contribute to attenuation of ZIKV and be applied to vaccine development across multiple platforms.

## 1. Introduction

Beginning in 2015, a Zika epidemic spread throughout the Americas, leading to congenital Zika syndrome. Currently, there is no licensed ZIKV vaccine, and candidate vaccines are in pre-clinical and clinical development [1,2]. ZIKV belongs to the Flaviviridae family, which includes other medically important mosquito-transmitted viruses such as yellow fever (YFV), dengue, Japanese encephalitis (JEV), and West Nile (WNV) viruses. Flaviviruses contain a positive-sense, single-stranded RNA genome that encodes a single polyprotein that is processed into 10 viral proteins. Three structural proteins—capsid, (pre)-membrane, and envelope—are present in the infectious, mature virion. Seven non-structural (NS) proteins (NS1, NS2A, NS2B, NS3, NS4A, NS4B, and NS5) have functions in viral replication and processing. Thus, molecular determinants present throughout the genome may affect flavivirus replication and pathogenesis.

Previous studies have shown that mosquito-transmitted flaviviruses could be attenuated by sequential passaging in HeLa cells. Studies in the 1960s showed that six serial passages of the virulent wild-type isolate YFV strain Asibi resulted in the virus losing viscerotropism in non-human primates (NHP) [3]. These studies were repeated and expanded in the 1990s by examining the YFV strain Asibi HeLa-passage 6 (P6) virus in mice, cynomolgus monkeys, and mosquitoes [4,5]. Monkey infection with wild-type YFV strain Asibi results in a lethal infection, whereas YFV strain Asibi HeLa-P6 virus infection resulted in survival. Further, YFV strain Asibi HeLa-P6 virus delays neurovirulence in suckling or weanling mice, reduces neuroinvasion in suckling mice, and leads to loss of mosquito competence in *Aedes aegypti* [4,5].

Mosquito-borne encephalitic flaviviruses have also been attenuated by passaging in HeLa cells. Specifically, WNV strain Sarawak HeLa-P6 virus was attenuated 4000-fold for mouse neuroinvasiveness compared to parental WNV. WNV strain Sarawak HeLa-P5 was also attenuated, but to a lesser extent [6]. Lastly, JEV strains Nakayama and 826309 HeLa-P6 viruses were both attenuated (3500-fold and 100-fold, respectively) in neuroinvasiveness and neurovirulence in adult mice. Interestingly, the JEV strain Nakayama was attenuated after a single passage in HeLa cells and remained attenuated through P6 and additional passage in other cell types [7]. Together, these studies showed that it was possible to attenuate mosquito-borne flaviviruses using serial passage in HeLa cells.

Therefore, we hypothesized that wild-type ZIKV human isolates would be attenuated after passaging in HeLa cells. ZIKV strain PA259249 survived the passage series and developed an attenuated phenotype. Additionally, the in-vitro-passaged ZIKV incorporated an amino acid substitution in both prM and NS1 proteins.

## 2. Materials and Methods 

### 2.1. Virus Passage

African green monkey kidney (Vero), human lung adenocarcinoma (A549), and human cervical cancer (HeLa) cells were acquired from American Type Culture Collection (ATCC )and maintained in minimum essential media (MEM) supplemented with 2 mM L-glutamine, 0.1 mM non-essential amino acids, 100 U/ml penicillin–100 μg/ml streptomycin, and 10% fetal bovine serum (viral passage media contained 2% FBS). Wild-type (WT) ZIKV strain FSS13025 (Cambodia, 2010) was passaged twice in Vero cells, and WT strain PA259249 (Panama, 2015) was passaged once in Vero cells to generate working stocks for the present study. HeLa cell monolayers were infected with ZIKVs at multiplicity of infection (MOI) of 0.1 and incubated for 4 days. Culture supernatant was then harvested and blind passaged onto fresh monolayers. ZIKVs underwent six passages in HeLa cells. Virus titers in the supernatants at each passage were titrated in Vero cells using plaque or focus formation assays. Vero cells were pre-seeded and incubated with 10-fold dilutions of virus stock for 30 minutes at room temperature prior to overlay with 1% agar-MEM, followed by incubation at 37 °C for three days. A second overlay containing 2% neutral red solution was added after three days, and plaques were visualized and counted the next day. Focus formation assays were performed in 12 well plates, with an overlay containing 0.8% carboxymethylcellulose (CMC) in MEM. After three days at 37 °C, the plates were fixed with acetone:methanol for 30 minutes and stained with mouse hyperimmune ascitic fluid against ZIKV (acquired from the World Reference Center for Emerging Viruses and Arboviruses, UTMB), followed by goat-anti-mouse antibody, and neutravidin-conjugated to HRP. Foci were developed with NovaRed substrate (Vector Labs). Only ZIKV strain PA259249 was viable after six passages, so all subsequent experiments were performed with the stocks from this series. Both plaque and focus formation assays resulted in the same viral titers; therefore, focus formation assays were used for subsequent experiments after HeLa cell passaging.

### 2.2. Evaluation of Viral Infection In Vitro

Replication of passaged viruses was evaluated in Vero and A549 cells. Cells were seeded overnight, and the following day were infected at MOI of 0.1 with WT ZIKV, HeLa-P4, or -P5 (or -P6 for Vero) viruses or with cell media (mock infection). Culture supernatants were harvested from Vero cells at 7 hours, and at 1, 2, 3, 4, and 5 days post infection (p.i.), and from A549 cells on days 1–3 p.i. Samples were titrated in Vero cells using focus formation assays (reported as focus forming units—ffu). For cytokine analysis, duplicate samples of infected A549 cells were gamma-irradiated to inactivate infectivity and analyzed with the Bio-Plex Pro Human Cytokine 27-Plex Assay (Bio-Rad) according to manufacturer’s instructions. Cytokine levels were compared using Kruskal–Wallis non-parametric test with Dunn’s post-test at each day p.i.

### 2.3. Mouse Experiments

Adult (8–12 weeks old) male and female AG129 mice (deficient in interferon α/β and γ receptor signaling) were inoculated via the intraperitoneal (i.p.) route with WT ZIKV (1 or 10 pfu) or P6 viruses. For HeLa-P6 infections, the results obtained from both 1 × 10^3^ (*n* = 2) and 2 × 10^3^ pfu (*n* = 7) doses were comparable and were combined for analysis, collectively referred to as 10^3^ pfu for reporting. To determine acute viremia and serum cytokine levels, blood was harvested by retro-orbital bleeds at 3 days p.i.; therefore, sera from all animals were examined. Mice were monitored for morbidity for up to 30 days p.i. Animals exhibiting signs of neurological disease were humanely euthanized according to approved protocols by the Institutional Animal Care and Use Committee of the University of Texas Medical Branch. At time of death (euthanasia), mouse brains and testes were harvested. Tissue samples were collected into pre-weighed tubes containing triple-pure 1.5 mm zirconium beads, weighed, homogenized in 1 milliliter (ml) of media in a BeadBug (Benchmark Scientific), and clarified via centrifugation. Viral loads in animal samples were titrated using focus formation assays, and titers are expressed as ffu per gram of tissue or ml of serum. Organs were harvested from a total of six mice (three female and three male mice) infected with 10^3^ pfu of HeLa-P6 for evaluation of six brain samples and three testis samples for viral titers. To determine the cytokine response, 3 day p.i. serum samples were γ-irradiated to inactivate infectivity and analyzed using the Bio-Plex Pro Mouse Cytokine 23-plex Assay (Bio-Rad) according to manufacturer’s intructions. Cytokine levels were compared using Kruskal–Wallis non-parametric test. To assess antibody induction, focus reduction neutralization tests (FRNT_50_) were performed using terminal mouse serum samples. Briefly, Vero cells were seeded overnight in 12 well plates. The following day, 50 pfu of WT ZIKV strain PA259249 virus were incubated with 2-fold dilutions of mouse serum for 30 minutes at 37 °C prior to addition to cells. Following incubation for 30 minutes, CMC overlay was added and focus formation assays were performed. After counting the foci in each well, the last dilution to neutralize at least 50% of the control infectivity (virus only wells) was determined to be the FRNT_50_.

### 2.4. Virus Sequencing 

To compare the sequences of passaged and unpassaged ZIKV genomes, viral RNA was extracted from cell culture supernatants using the QiaAmp Viral RNA Mini kit (Qiagen). HeLa-P1 through P6 genomes were sequenced using the Illumina next generation platform at the University of Texas Medical Branch NGS Core Laboratory. The TruSeq RNA v2 Library kit (Illumina) was used to construct cDNA libraries using random hexamers. Paired-end reads were processed as previously described [8] to remove poor-quality reads, adapter sequences, and duplicates and generate *de novo* sequences. The MUSCLE algorithm in MacVector with Assembler v17 was used to align *de novo* fasta consensus sequences of HeLa-P1–P6 to the publicly available sequence of ZIKV PA259249 (KX156775). A single replicate of HeLa-P2 and -P3 stocks and two replicates of HeLa-P1, -P4, -P5, and -P6 were sequenced and analyzed. To determine the sequence of viruses after AG129 mouse infection, viral RNA was extracted from homogenized tissue samples. Three brain and three testis samples from WT ZIKV-infected animals were sequenced and analyzed. From HeLa-P6-infected animals, six brain samples were sequenced (three from female and three from male, described above in “Mouse Experiments” subsection) and analyzed; two testis samples were sequenced, but only one was analyzed because the low viral titers in the testes of HeLa-P6-infected animals resulted in insufficient viral sequences in the samples. Sequences of the following ZIKVs were deposited in GenBank: PA259249 passaged in HeLa cells (accession number MN100039), PA259249 passaged in HeLa cells and AG129 mice (accession number MN124091), and PA259249 passaged in AG129 mice (accession number MN124090).

### 2.5. Structural and Schematic Renderings

ZIKV genome organization schematic was generated in MacVector v17. NS1 protein structure (PDB 5K6K) and prM-E cryo-EM protein structure (PDB 6CO8) were rendered using Pymol (v1.8.4.0). BioRender was used for the graphical abstract.

### 2.6. Statistical Analysis

Unpaired t-tests and non-parametric Mann–Whitney U tests; ANOVA, Kruskal–Wallis tests, and multiple comparison post-tests; and Mantel–Cox tests were determined using Prism v8.0 (GraphPad).

## 3. Results

### 3.1. Passaging of ZIKV PA259249 in HeLa Cells Led to In Vitro Attenuation 

**Figure 1:**
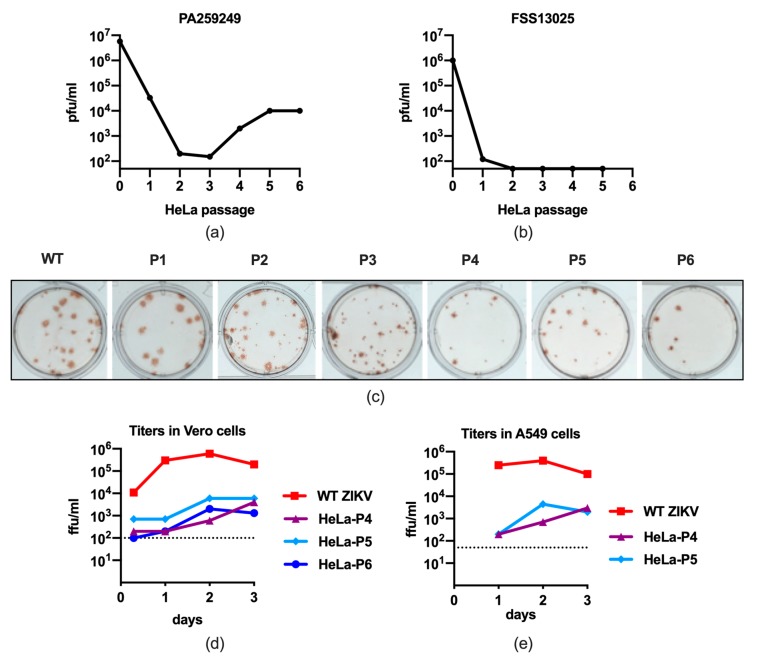
Passaging of ZIKV PA259249 in HeLa cells led to in vitro attenuation. ZIKV strain PA259249 (**a**) or FSS13025 (**b**) was serially passaged in HeLa cells up to six times (P6), and stocks were titrated in Vero cells. Titration limit of detection: 50 pfu (10^1.7^ pfu). (**c**) Focus morphology of passaged ZIKV PA259249; (**d**) stocks of WT Zika PA259249 virus serially passaged in HeLa cells, four, five, or six times (WT, HeLa-P4, -P5, and -P6, respectively) were used to infect Vero cells. Duplicate samples were harvested from cell supernatants at 7 hours, 1, 2, or 3 days p.i. and titrated in Vero cells. All titers decreased on days 4 and 5 (not shown). Dashed line: limit of detection: 100 ffu/ml (10^2.0^ ffu/ml). (**e**) Stocks of WT Zika strain PA259249 virus HeLa-P4 and -P5 were used to infect A549 cells; duplicate samples harvested from cell supernatants at 1, 2, or 3 days p.i. were titrated in Vero cells. Dashed line: limit of detection: 50 ffu/ml (10^1.7^ pfu/ml). Mock-infected cells had no detectable titers in Vero or A549 cells (not shown).

To determine whether ZIKV, like other flaviviruses, could be attenuated by passage in HeLa cells, two wild-type ZIKV strains, FSS13025 and PA259249, of the American/Asian lineage were passaged six times in HeLa cells (Figure 1a,b). Titers of ZIKV strain PA259249 in culture supernatants decreased rapidly until passage 3, but then slowly increased and stabilized at passages 5 and 6. Additionally, focus morphology became more uniform as the virus was passaged (Figure 1c). Ultimately, infectious titer following six passages of ZIKV strain PA259249 in HeLa cells was reduced by approximately 500-fold (Figure 1a). ZIKV strain FSS13025 failed to adapt for growth in HeLa cells (Figure 1b), and was not recovered during the passage series. Therefore, all subsequent experiments in the study were performed using WT ZIKV and HeLa-P1–P6 of strain PA259249.

The titers of passaged PA259249 viruses began to recover at HeLa-P4 and reached the highest titers at HeLa-P5 and -P6. Therefore, multiplication of the WT ZIKV, HeLa-P4, -P5, and -P6 viruses in Vero cells was determined. Viral loads following infection with HeLa-P4–P6 were consistently lower, by approximately 10^2^–10^3^ ffu/ml, than cells infected with WT ZIKV (Figure 1d). Regardless of infectivity, all titers had peaked by day 3 p.i. (days 4–5 not shown). Another multiplication curve was performed in the interferon-competent human epithelial cells, A549 (Figure 1e). Similarly to multiplication in Vero cells, HeLa-P4 and -P5 viruses replicated to significantly lower (up to 10^3^ ffu lower) titers at 1, 2, and 3 days p.i. when compared to WT ZIKV, and there was no significant difference in replication between HeLa-P4 and -P5 viruses (Figure 1e). Overall, the cytokine and chemokine response of these cells was consistent with the viral titers in the samples, in that A549 cells infected with WT ZIKV secreted higher levels than cells infected with HeLa-P4 or -P5 (Supplementary Figure S1). Specifically, pro-inflammatory cytokines and chemokines IL-6, CXCL8 (IL-8), IL-9, CCL4 (MIP-1β), CCL5 (RANTES), and CXCL10 (IP-10) were among those significantly elevated in WT-ZIKV-infected samples.

### 3.2. ZIKV Passaged in HeLa Cells Was Attenuated In Vivo and Generated an Immune Response

**Figure 2:**
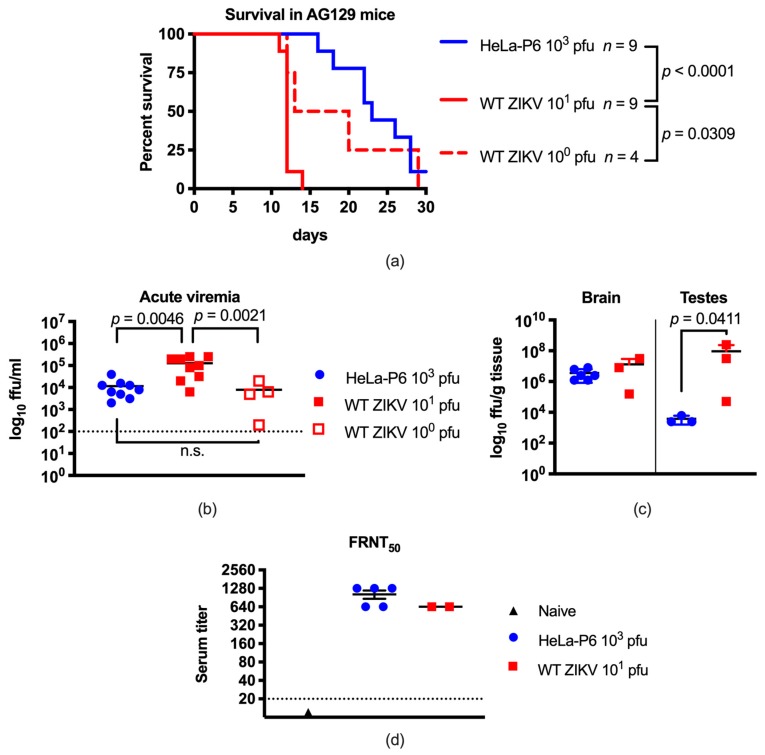
ZIKV passaged in HeLa cells was attenuated and generated an immune response in AG129 mice. (**a**) Adult AG129 mice were inoculated via the i.p. route with 10^0^ or 10^1^ pfu of WT ZIKV or with 10^3^ pfu of HeLa-P6 virus, and monitored for morbidity and mortality for 30 days. Statistics were determined using Log-rank (Mantel–Cox) tests. WT ZIKV 10^0^ pfu *n* = 4; WT ZIKV 10^1^ pfu *n* = 9; HeLa-P6 10^3^ pfu *n* = 9; (**b**) AG129 mice were bled 3 days p.i. to determine acute infection viremia levels. Lines indicate the mean titer. Statistics were determined using one-way ANOVA and Tukey’s post-test; n.s.: not significant. WT ZIKV 10^0^ pfu *n* = 4, WT ZIKV 10^1^ pfu *n* = 9, Hela-P6 10^3^ pfu *n* = 9; (**c**) At time of death, infected animal brain and testis samples were harvested to determine viral loads. Lines indicate means. Statistics were determined using *t* tests. WT ZIKV 10^1^ pfu days 11–12, brain *n* = 3, testis *n* = 3; HeLa-P6 10^3^ pfu brain *n* = 6, testis *n* = 3; (**d**) Neutralizing antibody titers present in terminal sera from euthanized mice infected with WT ZIKV 10^1^ pfu (*n* = 2, 11–12 days p.i.), HeLa-P6 10^3^ pfu (*n* = 5, days 16–27 p.i.) or naïve (*n* = 1) mice were determined using a FRNT_50_ assay in Vero cells. Endpoint titers are plotted in a log_2_ scale to reflect the 2-fold dilution series. Dashed line: limit of titer detection was 1:20. Results between WT ZIKV and HeLa-P6 were not significantly different by Mann–Whitney test.

To determine whether ZIKV HeLa-P6 had an attenuated phenotype in vivo, AG129 mice were infected with either 10^0^ or 10^1^ pfu WT ZIKV or 10^3^ pfu of HeLa-P6 and monitored for one month post infection (Figure 2a). All mice infected with both doses of WT ZIKV succumbed to infection, with animals inoculated with the higher dose dying between 11–14 days p.i. (median survival: 12 days), and those with the lower dose on days 12–29 (median survival: 16.5). Additional higher doses of WT ZIKV were not evaluated due to the rapid mortality induced by inoculation with 10^1^ pfu. HeLa-P6 also produced a universally lethal infection with the mice dying 16–28 days p.i. (median survival: 23), which differed significantly to the survival of mice infected with 10^1^ pfu of WT ZIKV (*p* < 0.0001), but not to that of mice infected with 10^0^ pfu of WT ZIKV. Because HeLa-P6 stock only reached titers of 10^4^ pfu, it was not possible to test an inoculum higher than 2 × 10^3^ pfu in the animals without subjecting the virus to purification steps that might affect virus biology.

Acute viremia in infected AG129 mice was determined at 3 days p.i. (Figure 2b). The mean acute viremia for animals infected with 10^1^ pfu of WT ZIKV was 10^4.9^ ffu/ml, which differed statistically from the viremia of animals infected with either 10^0^ pfu of WT ZIKV (10^3.5^ ffu/ml, *p* = 0.0021) or with 10^3^ pfu of HeLa-P6 (10^3.9^ ffu/ml, *p* = 0.0046). The viremia of infected AG129 mice followed a similar pattern to that observed for mouse survival, insomuch that infection with 10^3^ pfu of HeLa-P6 virus led to similar results as with 10^0^ pfu of WT ZIKV, but not as with 10^1^ pfu of WT ZIKV. Additionally, titer of infectious virus in the brain and testes at time of death was evaluated (Figure 2c). Viral load in the testes of mice infected with 10^1^ pfu of WT ZIKV (10^6.9^ ffu/g) was statistically higher than that of mice infected with 10^3^ pfu of HeLa-P6 (10^3.5^ ffu/g) (*p* = 0.0411). However, there was no statistical difference observed in the brain viral load, which is not unexpected given that all animals regardless of infection conditions succumbed to similar neurological disease. Ultimately, replication of HeLa-P6 was significantly attenuated in AG129 mice.

In order to determine whether infected AG129 mice generated antibodies against WT ZIKV, neutralization assays were performed. At time of death, terminal bleeds were performed to harvest serum. Mice infected with WT ZIKV 10^1^ pfu (days 11, 12 p.i.) had neutralizing antibodies against WT ZIKV with a titer of 1:640 (*n* = 2), and those infected with HeLa-P6 10^3^ pfu (days 16, 18, 22, 26, and 27 days p.i.) had neutralizing antibody titers of 640–1080 (*n* = 5) (Figure 2d). The titers of the two groups were not statistically different, indicating that inoculations with either WT ZIKV or HeLa-P6 resulted in production of neutralizing antibodies to WT ZIKV.

### 3.3. Infection with Attenuated ZIKV Led to an Elevated Immune Response

**Figure 3:**
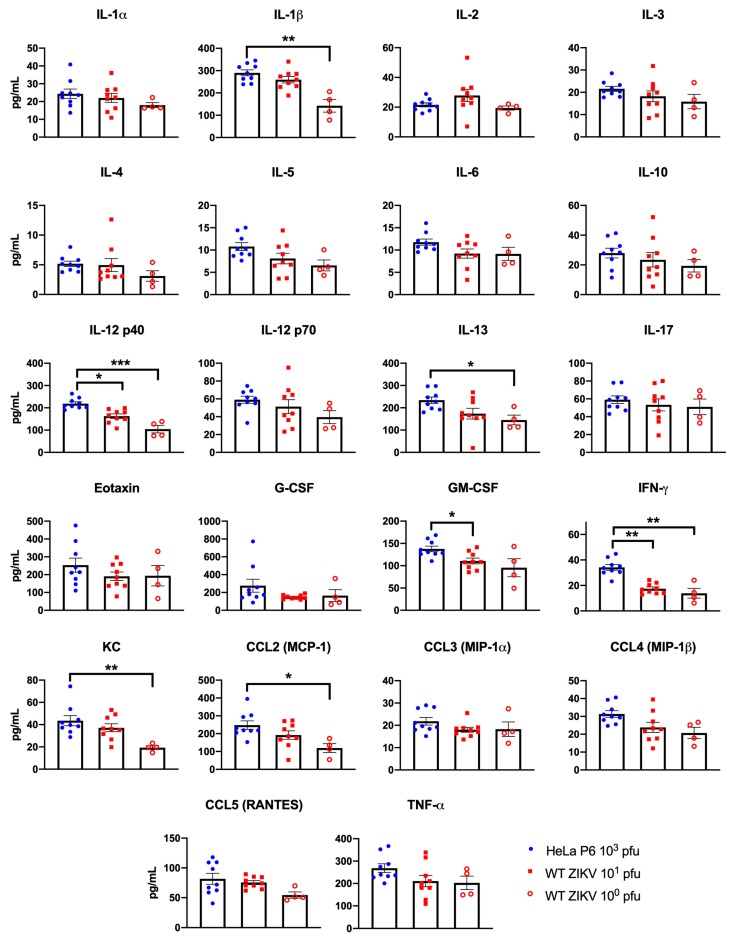
Passaging of ZIKV in HeLa cells increased circulating cytokines in AG129 mice. Mice were inoculated with WT ZIKV (10^0^ or 10^1^ pfu) or 10^3^ pfu HeLa-P6, and the circulating cytokine response was measured at 3 days p.i. Cytokine and chemokine levels increased following infection with HeLa-P6 compared to WT ZIKV. WT ZIKV 10^0^ pfu *n* = 4, WT ZIKV 10^1^ pfu *n* = 9, Hela-P6 10^3^ pfu *n* = 9. Kruskal–Wallis tests with Dunn’s multiple comparisons post-tests were used for statistical analysis. Bars represent the mean +/- standard error. * *p* < 0.05, ** *p* < 0.01, ** *p* < 0.001.

The cytokine response of mice inoculated with WT ZIKV or HeLa-P6 was evaluated at 3 days p.i. using Bio-plex. Mice inoculated with 10^3^ pfu HeLa-P6 consistently exhibited increased circulating cytokine levels compared to those inoculated with WT ZIKV (10^0^ or 10^1^ pfu) (Figure 3). Of the 23 cytokines evaluated, values were detected for all except IL-9. A total of seven cytokines were significantly increased in the sera of mice inoculated with HeLa-P6 compared to WT ZIKV (IL-1β, IL-12p40, IFN-γ, IL-13, GM-CSF, KC, and CCL-2). Only the Th1 cytokines IL-12p40 and IFN-γ were significantly increased in HeLa-P6-infected mice compared to mice infected with both doses of WT ZIKV. The pro-inflammatory cytokines and chemokines IL-1β, IL-13, KC, and CCL-2 were all significantly increased in the HeLa-P6-infected mice compared to the mice inoculated with 10^0^ pfu WT ZIKV. Further, GM-CSF was also significantly increased in HeLa-P6-infected mice compared to those inoculated with 10^1^ pfu WT ZIKV. These results indicated that the attenuated HeLa-P6 was capable of inducing an immune response in the infected mice during acute infection.

### 3.4. HeLa-Cell-Passaged ZIKV PA259249 Incurred Amino Acid Substitutions

**Figure 4:**
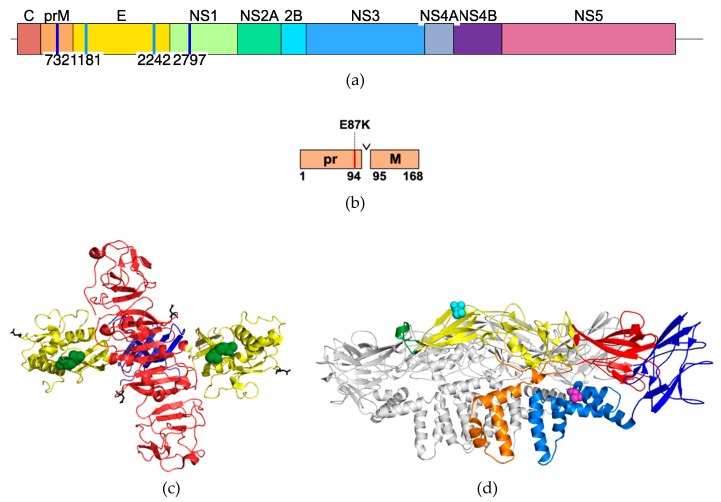
Location of amino acid substitutions incurred after HeLa cell passage**.** (**a**) ZIKV genome organization showing the non-synonymous nucleotide changes occurring after in vitro HeLa cell passaging at positions 732 and 2797 (dark blue vertical lines), and after in vivo mouse passaging of HeLa-P6 at 1181 and 2242 (light blue vertical lines). (**b**) Cartoon representation of the location of prM amino acid 87 in the pr peptide domain, which is cleaved by furin protease during maturation. (**c**) Top view of the NS1 protein dimer structure [9] shows the location of residue 103 (green spheres) in the wing domain (yellow). (**d**) Side view of of the cryo-EM [10] of three M–E proteins showing E 68 in cyan in domain 2 and E 422 in magenta in the stem region. One M and one E protein are shown in color (red—E domain 1, yellow—E domain 2, green—fusion loop, dark blue—E domain 3, marine blue—E stem and transmembrane, orange—M transmembrane).

To examine genetic differences among the unpassaged and passaged viruses, genome sequences were determined following each HeLa cell passage. Two amino acid substitutions were present in the genomes of HeLa-P3–P6 viruses when compared to WT ZIKV, HeLa-P1, and HeLa-P2 (Table 1). Nucleotide changes of genomic positions G732A and G2797A were identified and resulted in substitutions prM E87K and NS1 R103K, respectively. PrM E87K is located in the immature form of the protein present prior to cleavage by furin, and the NS1 R103K substitution is located in the external wing domain (Table 1 and Figure 4). One of two sequencing replicates for HeLa-P6 ZIKV had the reversion at NS1 R103; however, the sequences of two replicates of HeLa-P4 and HeLa-P5 had the substitution NS1 R103K.

Furthermore, virus harvested from the organs of infected mice (see Figure 2c) was subjected to sequencing in order to determine whether the acquired substitutions were present during in vivo inoculation. Both prM E87K and NS1 R103K amino acid substitutions were maintained following infection of AG129 mice with HeLa-P6 virus. The consensus sequence of virus recovered from brains (five of mice mice) and testes (one of one mouse) of HeLa-P6-infected AG129 mice contained the prM E87K and NS1 R103K amino acid substitutions. Additionally, three synonymous nucleotide substitutions G3767U, C5564A, and G5789A, and two non-synonymous substitutions G1181A (E M68I) and C2242U (E A422V) were common to the sequences of the HeLa-P6-infected animal-derived tissues (five of six brains and one testis sample) (Table 2 and Figure 4).

Other synonymous nucleotide substitutions were detected but were not shared among the tissue sample sequences (Table 2). Virus recovered from the brain tissue of one mouse infected with HeLa-P6 virus reverted and did not contain any amino acid substitutions common to the HeLa-passaged viruses, but, rather, contained three unique amino acid substitutions at E N207K, NS1 M349K, and NS3 A88T (Table 2). By comparison, three brain and three testis samples were sequenced from WT-ZIKV-infected AG129 mice. The virus sequences were identical to the parent unpassaged virus, with the exception of one common amino acid substitution of NS2A A117V (six of six samples) (Table 2). One unique synonymous nucleotide substitution was detected in the testis sample from a WT-ZIKV-infected mouse (Table 2). In summary, the two amino acid substitutions identified in the HeLa-P3–P6 viruses were retained in a majority (six of seven) of the HeLa-P6-infected animal-derived samples, and two additional common amino acid substitutions were acquired in vivo. Taken together with the in vitro and in vivo results of HeLa-passaged ZIKV, the amino acid substitutions at prM E87K and NS1 R103K may have contributed to the attenuation of ZIKV in vitro and in vivo, however, the role of the additional common amino acid substitutions at E M68I and E A422V is less clear, as they may play a role in attenuation or virulence of ZIKV in vivo.

## 4. Discussion

Initially, two human ZIKV strains, FSS13025 and PA259249, were passaged sequentially in HeLa cells. The ZIKV strain FSS13025 was rapidly lost during passaging. Results showed that ZIKV strain FSS13025 was too deficient in the initial passage and could not maintain sufficient replication to adapt. Titers of ZIKV strain PA259249 also rapidly decreased over the first two passages, but then slowly increased and stabilized by HeLa-P5, although there was approximately a 500-fold reduction in infectious virus, indicating that HeLa cell passage restricts ZIKV replication. This result is consistent with previous studies of HeLa passage attenuation of YFV and WNV, which also showed strain differences and reduced infectivity with early passage, followed by an increase in infectivity yields after HeLa-P3, indicating adaptation to HeLa cells [6,11]. Furthermore, multiplication curves in Vero cells were consistent with a deficiency of up to 10^3^ ffu in the HeLa-passaged stocks compared to the WT ZIKV stocks.

Multiplication kinetics of WT ZIKV strain PA259249 in A549 cells were similar to previous reports that showed ZIKV strains MR766 and PF-25013-18 replicate efficiently in A549 cells [12,13], while ZIKV PA259249 HeLa-P4 and -P5 viruses showed reduced replication in A549 cells, supporting the conclusion that the attenuated phenotype in vitro resulted from passaging ZIKV in HeLa cells. Additionally, in A549 cells, ZIKV PA259249 HeLa-P4 and -P5 viruses exhibited decreased multiplication kinetics. Moreover, A549 cells secreted higher levels of pro-inflammatory cytokines and chemokines following infection with WT ZIKV than with HeLa-P4 or -P5, presumably as a result of infectivity in the cells. Taken together, ZIKV was attenuated in vitro following passage in HeLa cells.

Consistent with previous studies of other wild-type ZIKV strains [14,15], infection of AG129 mice with 10^1^ pfu of WT ZIKV strain PA259249 caused significant viremia, replication in tissues, and fatality. However, reduced viremia and extended survival times were observed in mice infected with either 10^0^ pfu of WT ZIKV PA259249 or with 10^3^ pfu of HeLa-P6. Although different doses were used in this study, we estimated the in vivo attenuation of HeLa-P6 using survival statistics. Because 10^3^ pfu of HeLa-P6 differed in outcome to 10^1^ pfu of WT ZIKV (approximately 10^2^ pfu difference), but not to 10^0^ pfu WT ZIKV (10^3^ pfu difference), we estimate that HeLa-P6 was attenuated for mortality at least 100-fold; additional doses are needed to accurately determine the attenuation.

At time of death, viral loads in the brains of AG129 mice infected with WT ZIKV strain PA259249 (10^1^ pfu) or HeLa-P6 (10^3^ pfu) were comparable, suggesting that HeLa-P6 ZIKV retained neurotropism. However, viral titers in the testes of ZIKV HeLa-P6-infected mice were lower than in WT-ZIKV-infected mice, which may have been due to several reasons, including replication kinetics, time to death, and additional mutations. Taken together with the acute viremia results, attenuation of ZIKV PA259249 following HeLa cell passage may reduce dissemination and, possibly, sexual transmission of ZIKV; however, this can only be determined in future studies focusing on transmission of ZIKV.

The circulating cytokine response of mice inoculated with HeLa-P6 was increased compared to mice inoculated with WT ZIKV. Interestingly, the most significantly different cytokines were the classic Th1 cytokines IL-12p40 and IFN-γ , the induction of which may play a role in delaying disease in mice infected with HeLa-P6. However, pro-inflammatory cytokines and chemokines were also induced, which may contribute to the disease imparted by HeLa-P6 infection, ultimately causing the animals to succumb. The virus elicited cytokine responses that are important for attenuated vaccines. Furthermore, mice inoculated with either the WT ZIKV or HeLa-P6 generated neutralizing antibodies to WT ZIKV with FNRT_50_ titers ranging from 1:640–1:1280. These data indicate that the attenuated virus was capable of generating an equivalent neutralizing antibody response to that of WT ZIKV. Interestingly, it has been shown that all WT ZIKV strains, historical and contemporary, are considered to belong to a single serotype [16]. Therefore, antibodies that neutralize one ZIKV are expected to neutralize another.

HeLa-passage-attenuated YFV has been characterized. YFV strain Asibi-HeLa-P6 led to 10 amino acid substitutions located in the E (Q27H, D155A, M228K, K331R, H390P), NS2A (T48A), NS4B (I95M, V98I, E144K), and NS5 (P900L) proteins [11]. Further, the E protein substitutions led to a conformational change that mediated binding of the E protein monoclonal antibody H5 known to recognize only vaccine strains of YFV [11]. Ultimately, substitutions detected in the YFV Asibi-HeLa-P6 were not common to ZIKV strain PA259249 HeLa-P6, indicating a different adaptation process for ZIKV attenuation.

Considerable progress has been made in identification of residues that contribute to ZIKV tropism and virulence. In the present study, the substitutions prM E87K and NS1 R103K were identified in the attenuated ZIKV. PrM E87K is located in the immature form of the prM protein in close proximity to the furin cleavage site between amino acids 94 and 95 [17], so it is not clear what role it could play in maturation. The conserved NS1 R103K substitution is located in the α/β subdomain of the external wing domain, an epitope-rich domain that may mediate interactions with host proteins or other viral proteins during replication [[9,18,19]]. Examination of the dengue virus NS1 protein indicates that wing-domain amino acids participate in the replication of dengue virus [20,21]. Specifically, the connector subdomain is implicated in interaction with the NS4A-2K-4B cleavage intermediate and the β-ladder in secretion of NS1. Therefore, it is possible that ZIKV NS1 amino acid 103 functions in immunomodulation or replication, as observed in the dengue virus wing domain.

It is possible that both prM and NS1 substitutions are necessary for attenuation of HeLa-passaged ZIKV, as both arose at the same passage. However, it is not known if one substitution is compensatory for another. Future studies using a ZIKV PA259249 infectious clone would help to elucidate the role and contribution of each substitution to attenuation.

The results were in accordance with previous studies that indicated that genetic diversity and amino acid substitutions in both the prM and NS1 proteins may contribute to ZIKV virulence and fitness [22,23]. It was recently shown that the genetic diversity at the subconsensus sequence level of ZIKV strain PA259249 was increased in the prM and NS1 genes compared to other regions of the genome [23]. Moreover, the amino acid substitution S139N (prM S17N) of ZIKV was shown to increase infectivity and pathogenicity in vitro and in vivo, while a unique substitution at NS1 T233A of ZIKV was isolated from an infected fetus with clinical signs of microcephaly [24,25]. Further, NS1 V188 in ZIKV isolates is implicated in increased infectivity in mosquitoes and in antagonism of IFN-β induction [26,27]. 

In vivo, the virus derived from WT-ZIKV-infected mouse tissues incurred a NS2A A117V substitution. This substitution was previously detected as a minor variant in wild-type ZIKV strains DakAr41524, PA259249, PA259634, and THA/PL-Cal_ZV/2013; and the opposite substitution of V117A was a minor variant in ZIKV strains MEX-I-44/2016 and MYS-P6-740 [23,28]. This implies that both amino acids, alanine and valine, are present at NS2A 117 in varying frequencies, and infection of AG129 mice may select for valine.

Additional substitutions were also common to viruses (six of seven) recovered after AG129 mouse infection with ZIKV PA259249 HeLa-P6: E M68I and E A422V. It is possible that these two E protein substitutions provided fitness or genomic stability to the HeLa-P6 viruses in vivo. Further, amino acid E 68 is surface-exposed and could interact with the host during infection. Results also showed that HeLa-P6 reverted to a WT ZIKV genotype in one of the six mouse sequences evaluated. Therefore, this study showed that substitutions incurred during HeLa cell passage are attenuating but require additional genetic stability in vivo, which may lead to eventual mortality in AG129 mice.

Herein, an adapted virus was generated that was attenuated in a sensitive animal model of ZIKV, the AG129 mouse. Furthermore, the attenuated HeLa-P6 virus was immunogenic. During acute infection, the virus elicited Th1 cytokine responses. Additionally, animals inoculated with HeLa-P6 generated a neutralizing antibody response similar to the levels of neutralizing antibodies elicited by WT ZIKV virus. Oftentimes, several, multigenic mutations are needed in order to prevent live-attenuated viruses from reverting or causing disease. Therefore, the substitutions identified in this study could be investigated in a rationally-designed, live-attenuated ZIKV vaccine that incorporates attenuating mutations in other genes or domains to produce a stable, immunogenic, and safe ZIKV vaccine.

## 5. Conclusion

The present study shows that, like other mosquito-borne flaviviruses, ZIKV can be adapted in HeLa cells to become attenuated in vitro and in vivo in mice. Additionally, the results underscore the importance of incorporating sequence analysis in characterizing attenuated ZIKV in vivo, which has implications for rational vaccine design for ZIKV.

Studies of empirically-derived live-attenuated vaccine candidates for related flaviviruses, such as the YFV 17D and JEV SA14-14-2 vaccines, have shown that multiple attenuating mutations may be introduced into the genome as a result of passaging. Subsequent examination of these substitutions, individually or their domains, resulted in altered phenotypes [8,29,30]. Further, other candidate live-attenuated ZIKV vaccines have been designed by incorporation of attenuating substitutions identified in other flaviviruses, such as dengue virus and WNV [31,32]. To this end, a combinatorial approach in which several attenuating mutations identified in either the native or a related flavivirus can be rationally introduced in order to design a candidate live-attenuated ZIKV vaccine that is optimized for minimal virulence and maximal immunity. Thus, this multi-genic live-attenuated strategy to ensure safety and immunogenicity of flavivirus vaccines is proposed [33]. The current study has shown that substitutions in either prM, NS1, or both attenuated ZIKV, and has provided insight into gene regions that tolerate substitution, yet allow the proteins to remain immunogenic, similarly to what has been observed for other live-attenuated flavivirus vaccines.

Ultimately, this study has provided key information regarding attenuating mutations in ZIKV. Future studies will determine the contribution of each substitution to the attenuation of ZIKV. This strategy can be used in combination with other platforms or attenuating mutations located in different domains or genes to generate live-attenuated viruses with reduced virulence and increased immunity which can be applied to vaccine development. 

## Figures and Tables

**Table 1:** HeLa-passaged ZIKV PA259249 incurred two amino acid substitutions.

Position	Nucleotide Change	Codon Change	Amino Acid	Polyprotein Number	Protein Number	WT	P1 ^1^	P2	P3	P4 ^1,2^	P5 ^1^	P6 ^1^
732	G -> A	GAA->AAA	E -> K	209	prM 87	G	.	.	A	A	A	A
2797	G -> A	AGA->AAA	R -> K	897	NS1 103	G	.	.	A	A	A	A/G ^3^

^1^ Duplicate sequencing was conducted on HeLa-P1, -P4, -P5, and -P6. ^2^ One of two sequences of HeLa-P4 had a G to A silent mutation in nucleotide 10256 that was not detected in any other sequence in the entire study. ^3^ Denotes a difference in consensus sequences between two sequencing runs of the HeLa-P6 stock.

**Table 2:** Sequences of animal-derived viruses following infection with WT ZIKV or HeLa-P6 stocks.

							Brain			Testis			Brain						Testis
Position	Substitution	Codon Change	Amino Acid	Polyprotein Number	Protein Number	WT	WT_a_	WT_b_	WT_c_	WT_a_	WT_b_	WT_c_	P6	P6	P6	P6	P6_d_	P6	P6_d_
732	G->A	GAA -> **A**AA	E->K	209	prM 87	G	.	.	.	.	.	.	**A** ^1^	**A**	**A**	**A**	**A**	.	**A**
1181	G->A	AUG -> AU**A**	M->I	358	E 68	G	.	.	.	.	.	.	**A**	**A**	**A**	**A**	**A**	.	**A**
1598	U->A	AAU -> AA**A**	N->K	498	E 207	U	.	.	.	.	.	.	.	.	.	.	.	**A**	.
2242	C->U	GCA -> G**U**A	A->V	712	E 422	C	.	.	.	.	.	.	**U**	**U**	**U**	**U**	**U**	.	**U**
2797	G->A	AGA -> A**A**A	R->K	897	NS1 103	G	.	.	.	.	.	.	**A**	**A**	**A**	**A**	**A**	.	**A**
3535	U->A	AUG -> A**A**G	M->K	1143	NS1 349	U	.	.	.	.	.	.	.	.	.	.	.	**A**	.
3767	G->U	GUG -> GUU	V	1220	NS2A 74	G	.	.	.	.	.	.	U	U	U	U	U	.	U
3895	C->U	GCG -> G**U**G	A->V	1263	NS2A 117	C	**U**	**U**	**U**	**U**	**U**	**U**	.	.	.	.	.	.	.
4875	G->A	GCC -> **A**CC	A->T	1590	NS3 88	G	.	.	.	.	.	.	.	.	.	.	.	**A**	.
4913	G->A	GUG -> GTA	V	1602	NS3 100	G	.	.	.	.	.	.	.	.	.	A	.	.	.
5537	C->U	GGC -> GGU	G	1810	NS3 308	C	.	.	.	.	.	.	U	.	.	.	.	.	.
5564	C->A	GGC -> GCA	A	1819	NS3 317	C	.	.	.	.	.	.	A	A	A	A	A	.	A
5789	G->A	GAG -> GAA	E	1894	NS3 392	G	.	.	.	.	.	.	A	A	A	A	A	.	A
7592	U->C	AUU -> AUC	I	2495	NS4B 226	U	.	.	.	.	C	.	.	.	.	.	.	.	.
8647	G->A	AGG -> A**A**G	R->K	2847	NS5 327	G	.	.	.	.	.	.	.	.	**A**	.	.	.	.
10268	C->U	AAC -> AAU	N	3387	NS5 867	C	.	.	.	.	.	.	.	.	.	.	.	U	.

^1^ Table cell colors correspond to the common substitutions identified in viruses derived from WT-ZIKV-infected mice (pink: 3895) and HeLa-P6-infected mice (dark blue: 732, 2797 in the in vitro passage; light blue 1181, 2242, 3767, 5564, 5789 in the in vivo passage). Nucleotide substitutions leading to amino acid changes are in **bold**. Animal matched samples denoted with subscripts_a–d._

## Supplementary Materials

The following is available online at https://www.mdpi.com/2076-393X/7/3/93/s1, Figure S1: Passaging of ZIKV in HeLa cells attenuates cytokine production in A549 epithelial cells.
