# Supplementary material for: Attenuation of Zika Virus by Passage in Human HeLa Cells
Source: Vaccines (Basel). 2019 Aug 20;7(3):93. doi: 10.3390/vaccines7030093 (PMC6789458; doi:10.3390/vaccines7030093)
Supplement: Supplementary file 1 [file vaccines-07-00093-s001.pdf]

vaccines

MDPI

Attenuation of Zika Virus by Passage in Human HeLa Cells

Li Li [1], Natalie D. Collins [2,3], Steven G. Widen [4], Emily H. Davis [1], Jaclyn A. Kaiser [2], Mellodee M. White [5], M. Banks Greenberg [5], Alan D.T. Barrett [1,6,7], Nigel Bourne [2,5,6,7], Vanessa V. Sarathy [1,6,7,*]

[1] Department of Pathology, University of Texas Medical Branch, Galveston, TX 77555, USA; lili@utmb.edu (L.L.); emidavis@utmb.edu (E.H.D.); abarrett@utmb.edu (A.D.T.B.)

[2] Department of Microbiology and Immunology, University of Texas Medical Branch, Galveston, TX 77555, USA; natalie.d.collins.mil@mail.mil (N.D.C.); jakaiser@utmb.edu (J.A.K.); nibourne@utmb.edu (N.B.)

[3] Viral Disease Branch, Walter Reed Army Institute for Research, Silver Spring, MD 20910, USA

[4] Department of Biochemistry and Molecular Biology, University of Texas Medical Branch, Galveston, TX 77555, USA; sgwiden@utmb.edu (S.G.W.)

[5] Department of Pediatrics, University of Texas Medical Branch, Galveston, TX 77555, USA; m3white@utmb.edu (M.M.W.); mbgreenb@utmb.edu (M.B.G.)

[6] Sealy Institute for Vaccine Sciences, University of Texas Medical Branch, Galveston, TX 77555, USA

[7] Institute for Human Infections and Immunity, University of Texas Medical Branch, Galveston, TX 77555, USA

* Correspondence: vvsarath@utmb.edu; Tel.: 01-409-747-2403

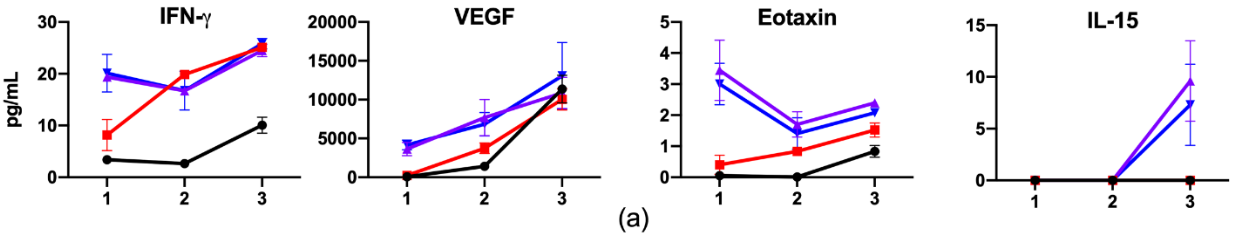
IFN-γ
VEGF
Eotaxin
IL-15
pg/mL
(a)

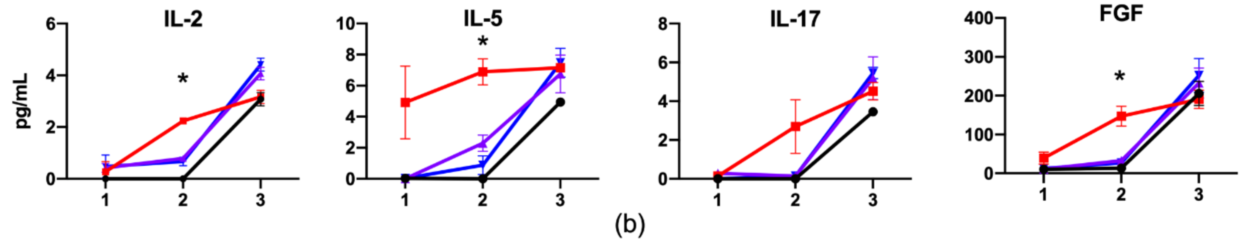
IL-2
IL-5
IL-17
FGF
pg/mL
*
(b)

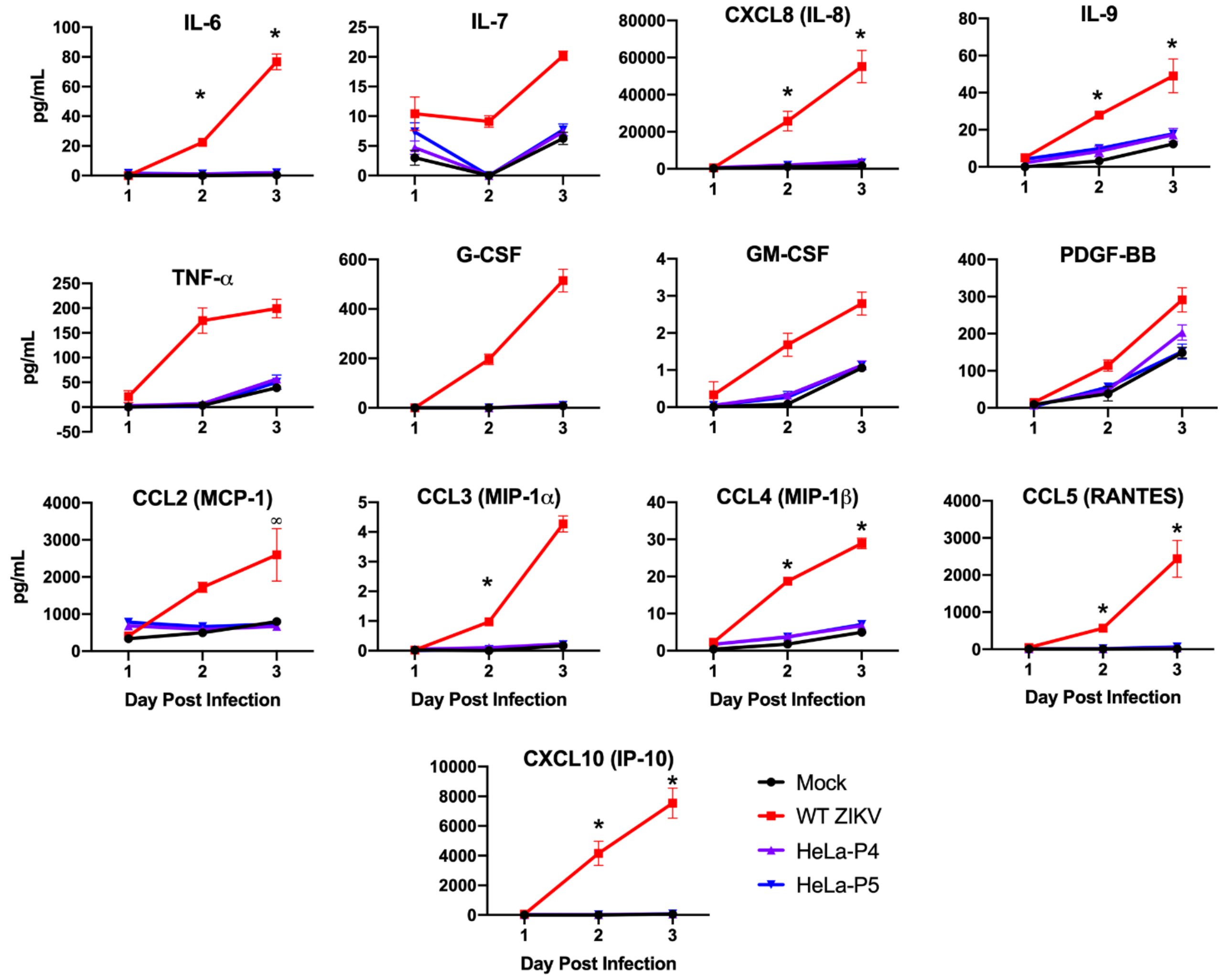
IL-6
IL-7
CXCL8 (IL-8)
IL-9
TNF-α
G-CSF
GM-CSF
PDGF-BB
CCL2 (MCP-1)
CCL3 (MIP-1α)
CCL4 (MIP-1β)
CCL5 (RANTES)
CXCL10 (IP-10)
pg/mL
Day Post Infection
Mock
WT ZIKV
HeLa-P4
HeLa-P5
(c)

Figure S1. Passaging of ZIKV in HeLa cells attenuates cytokine production in A549 epithelial cells. Secreted cytokine levels from A549 cells infected with WT ZIKV, HeLa-P4, HeLa-P5, or mock at 1, 2, or 3 days p.i were quantified from 50 microliter samples ($n = 2$). (**a**) Cytokine and chemokine levels increased on day 1, 2, or 3 following infection with HeLa-P4 and HeLa-P5 but not with WT ZIKV; (**b**) Cytokines and chemokines increased by infection with WT ZIKV on day 2 p.i. or (**c**) on days 2 and 3 p.i. Not shown: IL-1β, IL-4, IL-12p70, and IL-13 were detected at very low levels (< 2 pg/mL); IL-1RA and IL-10 were not detected in any samples. Kruskal-Wallis tests with Dunn's multiple comparisons were used to compare WT ZIKV, HeLa-P4, and HeLa-P5 to mock and to compare mock, HeLa-P4, and HeLa-P5 to WT ZIKV. Error bars represent standard deviation. *$p < 0.05$ WT ZIKV compared to mock; ∞ $p < 0.05$ WT ZIKV compared to HeLa-P4 or HeLa-P5. No differences were detected between HeLa-P4 and HeLa-P5 induction of cytokines.
